# Infectious Virions of *Bombyx Mori Latent Virus* Are Incorporated into *Bombyx Mori Nucleopolyhedrovirus* Occlusion Bodies

**DOI:** 10.3390/v11040316

**Published:** 2019-04-01

**Authors:** Keita Tsukui, Chihiro Yagisawa, Shota Fujimoto, Moe Ogawa, Ryuhei Kokusho, Mitsuyoshi Nozawa, Hideki Kawasaki, Susumu Katsuma, Masashi Iwanaga

**Affiliations:** 1 Department of Agrobiology and Bioresources, School of Agriculture, Utsunomiya university, Utsunomiya, Tochigi 321-8505, Japan; tki-kei@pref.gunma.lg.jp (K.T.); yagisanchihiro@gmail.com (C.Y.); s171397w@st.go.tuat.ac.jp (S.F.); nogaku.m74@gmail.com (M.O.); kawasaki@cc.utsunomiya-u.ac.jp (H.K.); 2 Department of Biological Production Science, United Graduate School of Agricultural Science, Tokyo University of Agriculture and Technology, Fuchu, Tokyo 183-8509, Japan; 3 Department of Agricultural and Environmental Biology, Graduate School of Agricultural and Life Sciences, The University of Tokyo, Bunkyo, Tokyo 113-8657, Japan; ryu.kokusho@gmail.com (R.K.); katsuma@ss.ab.a.u-tokyo.ac.jp (S.K.); 4 Institute of Sericulture, Ami, Ibaraki 300-0324, Japan; nozawa42@silk.or.jp

**Keywords:** *Bombyx mori latent virus*, nucleopolyhedrovirus, baculovirus, occlusion body, silkworm, persistent infection, Tymoviridae, BmLV, maculavirus, *Bombyx mori macula-like virus*

## Abstract

The *Bombyx mori latent virus* (BmLV) belongs to the unassigned plant virus family *Tymoviridae* and contains a positive-sense, single-stranded RNA genome. BmLV has infected almost all *B. mori*-derived cultured cell lines through unknown routes. The source of BmLV infection and the BmLV life cycle are still unknown. Here, we examined the interaction between BmLV and the insect DNA virus *Bombyx mori nucleopolyhedrovirus* (BmNPV). Persistent infection with BmLV caused a slight delay in BmNPV propagation, and BmLV propagation was enhanced in *B. mori* larvae via co-infection with BmNPV. We also showed that BmLV infectious virions were co-occluded with BmNPV virions into BmNPV occlusion bodies. We propose a new relationship between BmLV and BmNPV.

## 1. Introduction

*Maculavirus* (family Tymoviridae) is a plant virus genus typified by the *Grapevine fleck virus* (GFkV) [1]. The GFkV genome is 7654 base and comprised of a single-stranded RNA. It contains four putative open reading frames that encode an RNA-dependent RNA polymerase (*RdRp*), coat protein (*cp*), and one or two proline-rich proteins with unknown functions. The *Maculavirus* host range is restricted to the European and American *Vitis* species, but their vectors have not been identified. The *Bombyx mori macula-like virus* (BmMLV) is a positive, single-stranded RNA virus closely related to plant maculaviruses [2]. It was first identified from *B. mori* ovary-derived BmN4 cells, and its positive-strand RNA genome contains three putative genes: *rdrp*, *cp*, and *p15*. BmMLV is currently characterized as an unassigned *Tymoviridae*, and is designated as *Bombyx mori latent virus* (BmLV) [3]. Interestingly, *B. mori* larvae are not permissive to BmLV multiplication via hemocoelic injection, and BmLV RNA is not detected from mulberry leaves [2,4]. However, almost all *B. mori*-derived cell lines are contaminated with BmLV via an unknown infection route [5]. We established the first BmLV-negative *B. mori* cell line, BmVF, from *B. mori* embryos [5]. Unlike *Spodoptera frugiperda*-derived Sf9 cells, BmVF cells are permissive to persistent BmLV infection without strong cytopathic effects (designated as BmVF-MLV) [5,6]. Recently, we clarified that the abundance of BmLV transcripts in BmN4 cells is as much as 16% and revealed that BmLV RNA-derived small interfering RNAs (siRNAs) and PIWI-interacting RNAs (piRNAs) play essential roles in establishing persistent infection in *B. mori* cultured cells [7].

The Baculoviridae family, encompassing insect-specific DNA viruses, is comprised of large, double-stranded, circular DNA genomes, ranging from 80 to 180 k base pairs (bp), which are packaged in enveloped, rod-shaped virions. This viral family is divided into four genera *Alphabaculovirus*, *Betabaculovirus*, *Gammabaculovirus*, and *Deltabaculovirus*, which are lepidopteran-specific nucleopolyhedroviruses (NPVs), lepidopteran-specific granuloviruses (GVs), hymenopteran-specific NPVs, and dipteran-specific NPVs, respectively [8]. *Alphabaculoviruses* can be subdivided into group I or II NPVs according to phylogenetic studies [9]. During their life cycle, NPVs produce two virion phenotypes, budded virus (BV) and occlusion-derived virus (ODV). BVs are produced at the initial stage of the multiplication cycle, are responsible for systemic infection inside the insect host, and have a single nucleocapsid enveloped in a membrane, which is derived from budding from the host cell’s plasma membrane. ODVs are produced in the late stage of the cycle, are required for primary infection in the midgut epithelium cell of the insect host, and have one or multiple nucleocapsids enveloped in a membrane, which is derived from the host cell’s nuclear envelope [10]. Finally, mature ODVs are occluded in a protein matrix that forms polyhedra called occlusion bodies (OBs). OBs are liberated into the environment upon host cell liquefaction [11]. 

Recently, it has been reported that the OBs of *Spodoptera exigua multiple NPV* (SeMNPV) incorporate not only ODVs, but also *Spodoptera exigua iflavirus 1* (SeIV1: family *Ifulaviridae*) virions which replicate in the same hosts [12]. Large SeMNPV OBs incorporate small SeIV1 particles and may contribute to SeIV1’s stability in the environment. Co-infection with SeMNPV and SeIV1 was recently found to reduce host body weight and SeMNPV OB production significantly [13]. These findings suggested a relationship between the life cycles of NPVs and those of other small viruses. Thus, we hypothesized that there is an interaction between BmNPV OBs and BmLV. In this study, we analyzed the correlation between BmNPV and BmLV infections. BmNPV propagation was estimated in BmLV-positive and -negative cell lines. Additionally, the incorporation of BmLV particles into BmNPV OBs was examined, and the infectious potential of BmLV embedded in BmNPV OBs was assessed via viral inoculation assays.

## 2. Materials and Methods

### 2.1. Cell Lines, Viruses, Larvae, and Plaque Assays

BmN4 and Sf9 cells were maintained, as described previously, in TC-100 and IPL-41 media supplemented with 10% fetal bovine serum (FBS), respectively [14]. BmVF and BmVF-MLV cells [5] were maintained in a 50:50 (v/v) mixture of IPL-41 media supplemented with 10% FBS and Serum-free KBM720 media (Kohjin Bio, Saitama, Japan) at 25 °C. BmLV and the *Autographa californica multicapsid nucleopolyhedrovirus* (AcMNPV) C6 isolate were propagated in BmN4 and Sf9 cells, respectively [5,14]. Briefly, the BmLV solution was prepared as follows: 5 × 10^8^ BmN cells were homogenized in 20 mL of phosphate-buffered saline (PBS) and centrifuged at 6000 × g for 15 min at 4 °C. After centrifugation, the supernatant was concentrated with Amicon Ultra-15 Centrifugal filter 100K (Millipore, Billerica, USA) and was then filtered (0.22 μm-pore-size filter), and used as the virus solution [5]. The BmLV-free BmNPV T3 isolate was generated as described previously [15]. *B. mori* larvae (F1 hybrid N124 × C124) were reared on an artificial diet [16]. The cells were infected with BmNPV or AcMNPV at a multiplication of infection (MOI) = 10. Virus titers were expressed as plaque-forming units (PFUs) via plaque assays as described previously [14]. BmLV inoculation was performed as described previously [17]. Student’s *t*-test was performed with KaleidaGraph computer software version 4.1 (Synergy Software, Reading, PA, USA)

### 2.2. Co-Infection of *Bombyx mori latent virus* (BmLV) and *Bombyx mori nucleopolyhedrovirus* (BmNPV) in *B. Mori* larvae

On Day 1, fifth instar larvae were starved for several hours to avoid vomiting with ice anesthetization, injected with BmNPV (1 × 10^6^ PFU) and/or BmLV extracted from 1 × 10^6^ BmN4 cells, returned to an artificial diet, and maintained at 25 °C. For reverse transcription-quantitative polymerase chain reaction (RT-qPCR) analysis, the total RNA was isolated from the fat bodies at designated times by RNAiso Plus (Takara Bio, Shiga, Japan). First-strand cDNA was synthesized to serve as qPCR templates using a Verso cDNA Synthesis Kit (Thermo Fisher Scientific, Waltham, MA, USA). Subsequently, cDNA fragments were subjected to qPCR analysis using primers BmLV-3361_3454-L (5′-CTGGTACGGCACCACTCTTT-3′) and BmLV-3361_3454-R (5′-ATGAGTTCCATGCCTCCAAG-3′) for BmLV *RdRp*, orf78 qPCR F (5′-CCCTGTGGATTTGTTCGTGTTTGACCC-3′) and orf78 qPCR R (5′-CCACAGTACCGTCGGCCATTTTGAG-3′) for BmNPV orf78 (*P143*) and using the SYBR Premix Ex Taq II (Takara Bio) and LightCycler 96 (Roche, Basel, Switzerland). Student’s *t*-test was performed with KaleidaGraph.

### 2.3. Detection of BmLV from BmNPV Occlusion Bodies (OBs)

BmLV-positive BmNPV OBs were extracted from BmNPV-infected BmN4 cells, as described previously [16]. To prepare the BmLV-negative BmNPV OBs, fifth instar larvae were injected with BmLV-free BmNPV (1 × 10^6^ PFU) on Day 1. At 96 hpi, the infected larvae’s hemolymph was collected, and the OBs were extracted using 0.1% SDS, as described previously [16]. To investigate whether BmLV was incorporated in BmNPV OBs, BmLV-positive and -negative OBs were dissolved in 5 × alkaline solution [18], electrophoresed, transferred on a NitroBind Transfer Membrane (GVS, Zola Predosa, Bologna, Italy), and probed with either BmLV CP [5], *B. mori* HEAT SHOCK COGNATE 70-4 [19], or beta-ACTIN antibodies (sc-1616, Santa Cruz Biotechnology, Santa Cruz, CA, USA). The anti-rabbit IgG, conjugated to horseradish peroxidase (KPL, Gaithersburg, MD, USA), and Western blotting substrate plus (Thermo Fisher Scientific) were used. Signal detection was carried out using the LightCapture II system (ATTO, Tokyo, Japan). For virus adhesion assays, 3 × 10^8^ BmLV-negative OB suspension was incubated with BmLV extracted from 4 × 10^5^ BmN4 cells for 60 min at 25 °C. After centrifugation at 3000 × *g* for 10 min, OBs were washed twice with 0.01% SDS and rinsed three times with PBS. OB dissolution and Western blotting was performed as described above. For electron microscopic analysis, the OBs were purified from BmVF or BmN4 cells infected with BmNPV and fixed using 2% glutaraldehyde in PBS. The OBs were then fixed using 1% osmium tetroxide in PBS overnight, dehydrated using a standard acetone series, and embedded in epoxy resin. The 70-nm sections were stained with uranyl acetate and lead citrate and viewed using a JEM-2000EX transmission electron microscope (JEOL, Tokyo, Japan) operated at 200 kV.

### 2.4. Infection of BmLV Incorporated in BmNPV OBs

To investigate whether the BmLV incorporated in OB was infectious, 1 × 10^5^ BmLV-positive and -negative OBs were dissolved in 5 × alkaline solution and then neutralized by 0.1 N HCl. After filtration using a 0.22 µm syringe filter, the OB solutions were inoculated into 4 × 10^6^ BmVF cells. For RT-PCR analysis, total RNA was extracted at 0 and 96 hpi using RNAiso plus. First-strand cDNA was synthesized using a Verso cDNA Synthesis Kit. Subsequently, cDNA fragments were subjected to RT-PCR analysis using primers MLVcp-5346-F (5′-TTTCTGCCGCTTCGGCCATTCCCTCCTTG-3′) and MLVcp-6007-R (5′-AGATACGCTGATGGAGCCTCTGATGACAACG-3′) for BmLV *cp*, polh1-F (5′-CCACCATCGGGCGTACTTACGTGTACGAC-3′) and polh711-R (5′-CGCGTCTGGTGCAAACTCCTTTATTTTGAAAAC-3′) for BmNPV *polyhedrin* (*polh*), and BA3F1 (5′-AGATGACCCAGATCATGTTCG-3′) and BAR1 (5′-GAGATCCACATCTGTTGGAAG-3′) for *B. mori actin3* [2] as well as using KAPATaq Extra (KAPA BIOSYSTEMS, Woburn, MA, USA). To evaluate the infectious potential of BmLV incorporated into *B. mori* larval OBs, fourth instar larvae were fed with small blocks of an artificial diet containing 1 × 10^7^ BmLV-positive OBs on Day 1. At 96 hpi, newly produced OBs were extracted from larval hemolymph, and were dissolved in an alkaline solution. After neutralization, the OB lysate was inoculated into BmVF cells. Detection of BmLV *cp* RNA using RT-PCR was performed as described above. We also examined whether BmLV was embedded in OBs collected from sericultural farms. The OBs were isolated from many cadavers from two sericultural farms (Nasushiobara, Tochigi, Japan; Yasato, Ibaraki, Japan). Dissolution of OB, inoculation of OB lysate, and detection of BmLV *cp* RNA were performed as described above. 

## 3. Results and Discussion

### 3.1. Persistent Infection with BmLV Affects the Multiplication of BmNPV in *B. Mori* Cultured Cells

**Figure 1:**
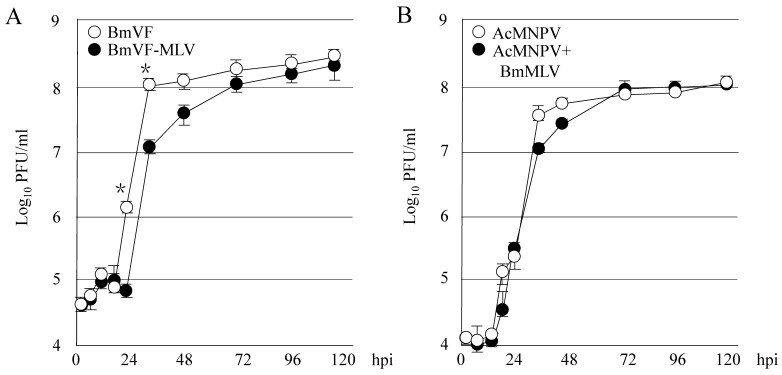
Growth curves of *Bombyx mori nucleopolyhedrovirus* (BmNPV) and *Autographa californica multicapsid nucleopolyhedrovirus* (AcMNPV). (**A**) Comparison of BmNPV propagation in *Bombyx mori latent virus* (BmLV)-negative BmVF (open circles) and -positive BmVF-MLV (closed circles) cells. The cells were inoculated with BmNPV at an MOI of 10. At 2, 6, 12, 18, 24, 36, 48, 72, 96, and 120 hpi, culture supernatant was collected and subjected to a plaque assay on BmN4 cells (mean ± s.d., *n* = 3, * *p* < 0.05). (**B**) Comparison of AcMNPV propagation in Sf9 cells in the presence or absence of BmLV. The cells were inoculated with AcMNPV or co-inoculated with AcMNPV and BmLV. At 2, 6, 12, 18, 24, 36, 48, 72, 96, and 120 hpi, culture supernatant was collected and subjected to a plaque assay on Sf9 cells (mean ± s.d., *n* = 3).

To investigate whether persistent infection with BmLV affects BmNPV proliferation, BmLV-negative BmVF or -positive BmVF-MLV cells were inoculated with BmNPV. As shown in Figure 1A, it was observed that the BmNPV proliferation in BmVF-MLV cells was less than that in BmVF cells at 24 and 36 hpi. The multiplication of BmNPV plateaued at 36 and 72 hpi in BmVF and BmVF-MLV cells, respectively. Next, to investigate the effects of the inoculation with BmLV on NPV multiplication, *S. frugiperda*-derived Sf9 was inoculated with both AcMNPV and BmLV or AcMNPV alone. As shown in Figure 1B, co-inoculation with BmLV in Sf9 slightly delayed the growth of AcMNPV at 36 and 48 hpi. We have reported that Sf9 are not permissive to BmLV persistent infection but approximately 60% of virus RNA remained at 7 days postinfection compared to just after inoculation [6]. These results indicated that persistent infection or co-inoculation with BmLV is involved in the multiplications of these NPVs. Host gene expression is either up- or down-regulated by infection with BmNPV or BmLV [7,20,21]. The host cell environment, changed upon BmLV infection, may have affected BmNPV proliferation. Additionally, we reported previously that both siRNA and piRNA pathways in *B. mori*-derived cells are responsible for suppressing BmLV proliferation [7]. Therefore, activation of these pathways, caused by BmLV infection, may delay the multiplication of these NPVs. Interestingly, the interaction between the persistently infective virus *Aedes albopictus densovirus* (AalDNV) and lethally infectious *Dengue virus serotype 2* (DEN-2) in *A. albopictus*-derived C6/36 cells illustrated that AalDNV infection significantly decreased DEN-2 proliferation [22]. Regulation of host gene expression and/or activation of host defense by AalDNV persistent infection may have reduced DEN-2 proliferation.

### 3.2. Co-Infection with BmLV and BmNPV Causes Proliferation of BmLV in *B. Mori* larvae

**Figure 2:**
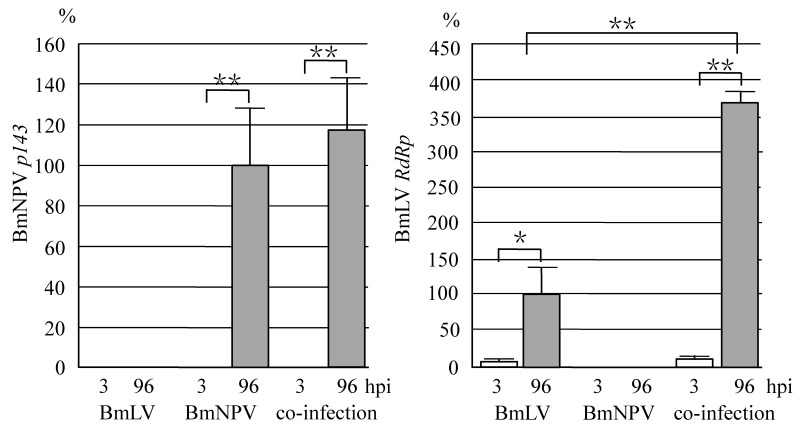
BmLV propagation in *B. mori* larvae is enhanced by co-infection with BmNPV. On Day 1, fifth instar larvae were injected with BmLV, BmNPV, or co-injected with both. At 3 (open column) and 96 hpi (closed column), RNA was extracted and subjected to reverse transcription-quantitative polymerase chain reaction (RT-qPCR) using primers for BmLV *RdRp* and BmNPV *p143* (mean + s.d., *n* = 3, * *p* < 0.05, ** *p* < 0.01).

We previously reported that BmNPV multiplicated vigorously in *B. mori* larvae, whereas BmLV did not replicate in *B. mori* larvae [2]. To investigate the interaction between BmLV and BmNPV in vivo, *B. mori* larvae were injected hemocoelically with BmLV, BmNPV, or both. As shown in Figure 2, while an abundant expression of BmNPV *p143* was observed in the fat body of BmNPV-infected larvae, no *p143* RNA was detected in BmLV-infected larvae. There was no significant difference in the amount of *p143* RNA between BmNPV-injected and co-injected larvae. On the other hand, while no BmLV *RdRp* was expressed in BmNPV-injected larvae, obvious *RdRp* RNA was observed in BmLV- and co-injected larvae (Figure 2). Interestingly, the *RdRp* RNA expressed in the larvae co-injected with BmLV and BmNPV increased approximately 3.7-fold as compared to that injected with BmLV alone. This result suggested that a productive infection of BmLV can be enhanced in *B. mori* larvae through the replication of BmNPV. Unlike cultured cells, each larval tissue of *B. mori* is covered with a basement membrane, which is an extracellular matrix. Since NPV infection causes degradation of the basement membrane due to the sufficient release of viral particles, BmLV may have readily attached to the host cells of *B. mori* larvae infected with BmNPV.

### 3.3. BmLV Infectious Virions are Incorporated into OBs of BmNPV

**Figure 3:**
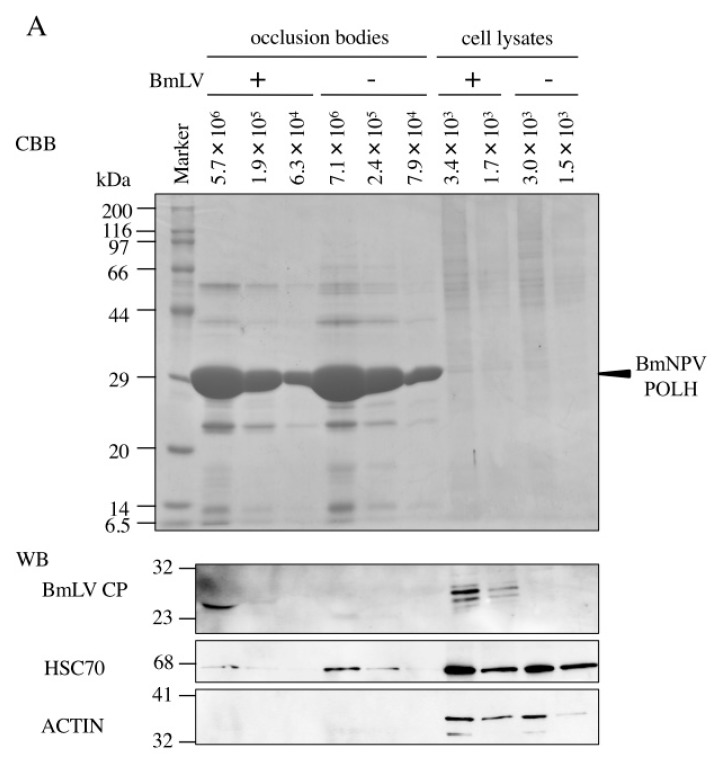

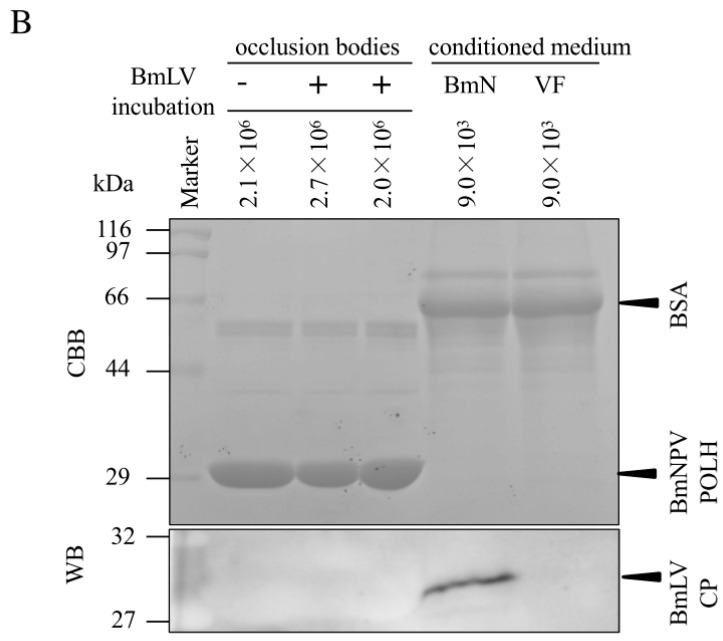
BmLV is incorporated into BmNPV occlusion bodies (OBs). (**A**) Western blot analysis of BmNPV OBs. BmNPV OBs from BmLV-positive and -negative cells were subjected to sodium dodecyl sulphate-polyacrylamide gel electrophoresis (SDS-PAGE), followed by Western blotting using anti-BmLV coat protein (CP), BmHSC70-4, and beta ACTIN antibodies. BmLV-positive BmN4 and -negative BmVF cell lysates were used as controls. The arrowhead indicates BmNPV POLH. Size markers are indicated on the left side of the panel. (**B**) BmLV adhesion assay to BmLV-negative OBs. After one hour of incubation with BmLV, OBs were washed and subjected to SDS-PAGE, followed by Western blotting using an anti-BmLV CP antibody. The arrowheads indicate bovine serum albumin (BSA), BmNPV POLH, and BmLV CP. Size markers are indicated on the left side of the panel.

**Figure 4:**
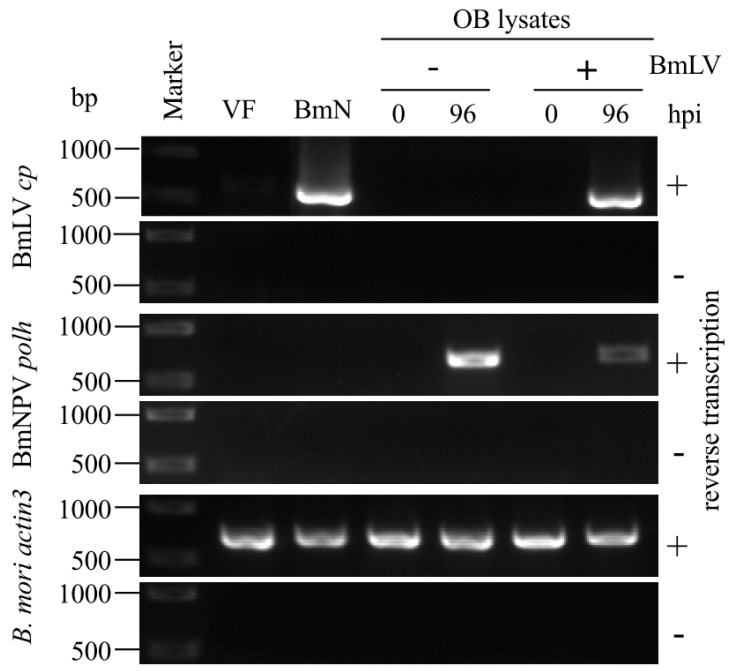
Infection study of BmNPV OB lysates. BmLV-negative and -positive OBs were dissolved in an alkaline solution and inoculated onto BmVF cells. At 0 and 96 hpi, RNA was extracted and subjected to RT-PCR using primers for BmLV *cp*, BmNPV *polh*, and *B. mori actin3*. BmLV-negative BmVF and -positive BmN4 cells were used as controls. Size markers are indicated on the left side of the panel.

Jakubowska et al. have reported a close relationship between SeMNPV and another small RNA virus, SeIV1, replicating in the same host [12]. The SeMNPV OB incorporates SeIV1 virions, and co-infection with SeMNPV and SeIV1 results in reduction of both host body weight and SeMNPV OB production [12,13]. To investigate whether BmNPV OBs incorporate BmLV particles, BmNPV OBs from BmLV-positive and -negative cells were subjected to Western blot analysis using BmLV CP antiserum. As shown in Figure 3A, while there was no detectable signal from the OBs prepared from *B. mori* larvae, BmLV CP signals were observed in the OBs prepared from BmN4 cells. While the signal intensity of HSC70 (a structural protein of ODV detected from OBs) is weaker than that from cultured cells, CP signal intensity detected in 5.7 × 10^6^ OBs was comparable to that in 3.4 × 10^3^ BmLV-positive cells (Figure 3A). In addition, while several CP signals were observed in BmLV-positive cultured cell lysates, one specific CP signal was detected from BmLV-positive OBs. Our previous study showed that several CP signals were observed in BmLV-infected cell lines, and one specific CP signal was detected from the culture medium containing infectious BmLV particles released from BmLV-infected BmVF cells [5]. These results suggested that this CP signal detected from OBs from BmLV-positive cells was derived from mature BmLV particles embedded into BmNPV OBs. Next, to exclude the possibility that the CP signal detected from OBs was due to the attachment of BmLV virions to the OB surface, 3 × 10^8^ OBs with no CP signal detected were incubated with a conditioned medium prepared from 4 × 10^5^ BmN4 cells, including an abundant amount of mature BmLV. As shown in Figure 3B, no CP signal was observed in either 2.7 × 10^6^ or 2.0 × 10^6^ OBs incubated with BmLV, while a clear CP signal was detected from the conditioned medium of 9.0 × 10^3^ BmN4 cells. Also, to detect the BmLV particles embedded in OBs, the OBs from BmLV-positive and -negative cells were observed using transmission electron microscopy. Interestingly, although it is unclear whether it is BmLV, some ambiguous particles were observed in OBs from BmLV-positive cells but not in those from BmLV-negative cells (Supplementary Figure S1). These results suggested that BmLV virions are incorporated into the inside of BmNPV OB. Next, to clarify whether BmLV particles incorporated into OBs were infectious, BmLV-positive and -negative OBs were dissolved in an alkaline solution; then, these OB lysates were inoculated into BmLV-negative BmVF cells. As shown in Figure 4, at 96 hpi, while no amplified product of *cp* was detected in BmVF cells inoculated with the lysate of BmLV-negative OBs, a clear *cp* band was observed in that of BmLV-positive ones. As a control, we examined the expression of BmNPV *polh* in infected cells. Although ODVs are much less infectious to cells compared to BVs [23], the amplified products of *polh* were detected from BmVF cells inoculated with either BmLV-positive or -negative OB lysates (Figure 4). These results indicated that small numbers of BmLV infectious virions were incorporated in BmNPV OBs.

### 3.4. Incorporation of BmLV into BmNPV OBs Is not Maintained in *B. Mori* larvae

**Figure 5:**
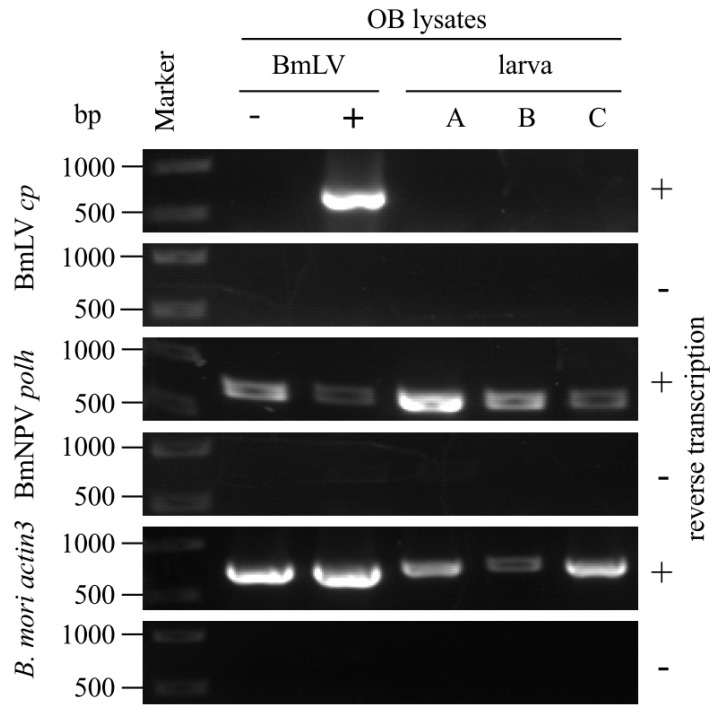
Infection study of the OB lysates collected from *B. mori* larvae co-infected with BmNPV and BmLV. On Day 1, fourth instar larvae were orally infected with BmLV-positive OBs. At 96 hpi, OBs were extracted from the hemolymph of three larvae (A–C) and dissolved in an alkaline solution. The OB lysates were inoculated onto BmVF cells. At 96 hpi, RNA was extracted and subjected to RT-PCR using primers for BmLV *cp*, BmNPV *polh*, and *B. mori actin3*. BmLV-negative and -positive OB lysates were used as controls. Size markers are indicated on the left side of the panel.

To investigate whether BmLV particles are incorporated into OBs in *B. mori* larvae as well as cultured cells, BmLV-positive OBs were administered orally to *B. mori* larvae. The newly produced OBs were collected from larval hemolymph, dissolved, and inoculated into BmLV-negative BmVF cells. As shown in Figure 5, no detectable *cp* band was observed in BmVF cells inoculated with OB lysates collected from each of three larvae (A–C). In addition, BmLV was not incorporated into BmNPV OBs collected from two sericultural farms (Supplementary Figure S2). These results showed BmLV was incorporated into BmNPV OBs in BmLV-positive cultured cells but not *B. mori* larvae. In our previous study, *B. mori* ovary-derived BmN cells were persistently infected with abundant BmLV, and RNA-seq analysis demonstrated that virus RNA in this cell line reaches 16% of the total RNA [2,7]. On the other hand, BmLV RNA did not increase clearly in *B. mori* larvae by hemocoelic inoculation [2]. These results suggested that the amount of BmLV proliferation in host cells is important for the incorporation of BmLV into BmNPV OBs.

Is the life cycle of BmLV associated with that of BmNPV? A previous study showed that almost all *B. mori*-derived cell lines were contaminated with BmLV through unknown routes [5]. However, BmLV has not been detected in other insect-derived cell lines and does not propagate in *S. frugiperda*-derived Sf9 or eight mammalian-derived cell lines [2,5,6]. This indicates that BmLV is a *B. mori*-specific virus. In this study, BmLV could propagate in *B. mori* larvae via co-infection with BmNPV (Figure 2). In cultured cells persistently infected with BmLV, BmLV infectious particles were incorporated into BmNPV OBs (Figure 3 and Figure 4). These results suggested that the lifecycle of BmLV in cultured cells is involved in that of BmNPV. On the other hand, BmLV incorporation was not maintained in *B. mori* larvae, and BmLV was not detected from OBs collected from sericultural farms (Figure 5 and Supplementary Figure S2). These results showed that the life cycle of BmLV in *B. mori* larvae is not correlated with that of BmNPV. Interestingly, recent works showed that plant tymovirus-like insect RNA viruses showing close homology to BmLV are widely present not only in *B. mori* cells but also in honeybee, bumblebee, and mite [24,25]. It is possible that these insect RNA viruses interact with other viruses co-infected in the same individual.

In conclusion, the present study determined, for the first time, that a plant virus-like insect RNA virus is incorporated into the occlusion bodies of an insect DNA virus and possesses infectious properties. We identified that the persistent or acute infection with BmLV affected BmNPV multiplication, and we found BmLV propagation in *B. mori* larvae was enhanced by co-infection with BmNPV (Figure 1 and Figure 2). We observed that BmLV infectious virions were incorporated into BmNPV OBs produced in *B. mori*-derived cultured cells (Figure 3 and Figure 4). Future research is warranted to understand whether this correlation also occurs in other plant virus-like insect RNA viruses and other insect DNA viruses.

## Supplementary Materials

The following are available online at https://www.mdpi.com/1999-4915/11/4/316/s1, Figure S1: Electron microscopic analysis of BmNPV OBs, Figure S2: Infection study of OB lysates collected from sericultural farms.
