# Supplementary material for: Infectious Virions of Bombyx Mori Latent Virus Are Incorporated into Bombyx Mori Nucleopolyhedrovirus Occlusion Bodies
Source: Viruses. 2019 Apr 1;11(4):316. doi: 10.3390/v11040316 (PMC6521139; doi:10.3390/v11040316)
Supplement: Supplementary file 1 [file viruses-11-00316-s001.pdf]

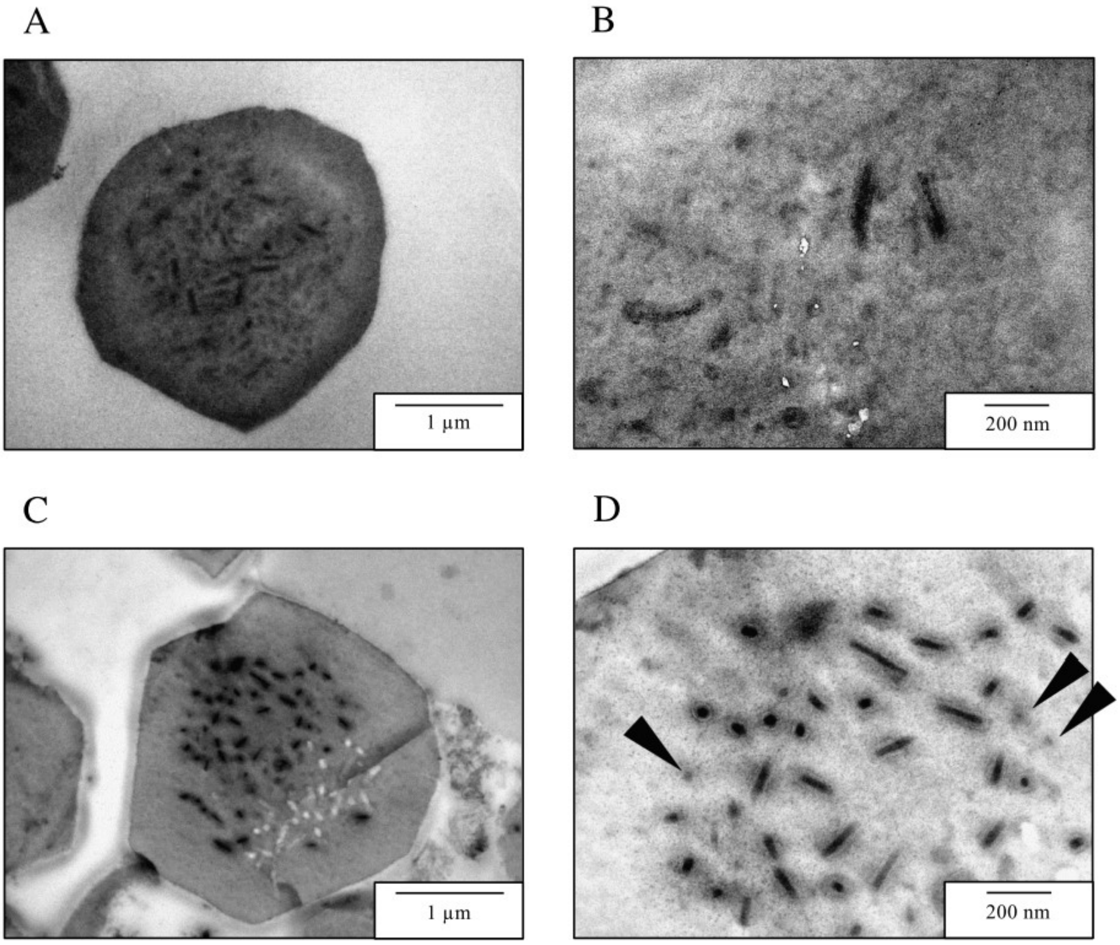

Figure S1: Electron microscopic analysis of BmNPV OBs. Representative images of BmLV-negative (**A**,**B**) and -positive (**C,D**) OBs. The OBs were purified from BmVF (A,B) or BmN4 (C,D) cells infected with BmNPV, followed by transmission electron microscopic analysis. Arrowheads indicate ambiguous particles. Scale bars represent 1 μm and 200 nm.

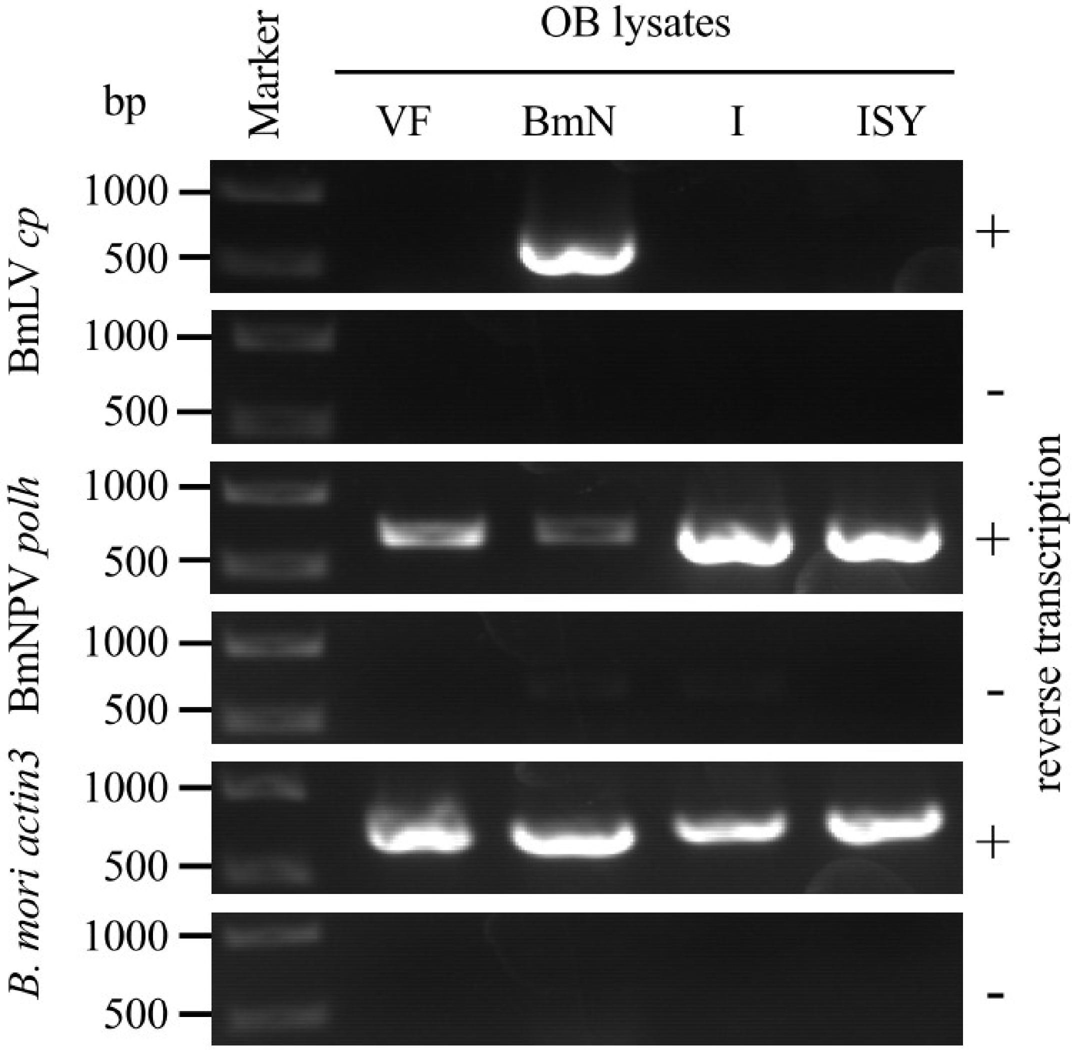

Figure S2: Infection study of OB lysates collected from sericultural farms. The OBs were isolated from several cadavers from two sericultural farms (I: Nasushiobara, Tochigi, Japan; ISY: Yasato, Ibaraki, Japan). OBs were dissolved in an alkaline solution, and then the OB lysates were inoculated onto BmVF cells. At 96 hpi, RNA was extracted and subjected to RT-PCR using primers for BmLV cp, BmNPV polh, and B. mori actin3. BmLV-negative BmVF and -positive BmN4 cells were used as controls. Size markers are indicated on the left side of the panel.
